# Thyroid Gland

**Published:** 1905-11-11

**Authors:** 


### THYROID GLAND.

**On the Nature and Treatment of Diseases of the Thyroid Gland.**—The result of recent careful examination [1] of cases of myxœdema and Graves disease tends to show that actual pathological changes of a primary nature are confined to the thyroid gland itself, and that although the nervous system in both cases is so profoundly affected, yet it is not the seat of any organic change. This fact shows that the nervous phenomena are caused by a toxic agent, and the manner in which they may completely disappear under treatment supports this view. Nevertheless in extreme cases of Graves disease, when protrusion of the eyes has led to corneal ulceration, operations on the cervical sympathetic may be of benefit, and Tomaselli [2] reports favourable results in such a case when he removed the middle and lower cervical ganglia on the most affected side. But the main direction of research in these diseases lies in the elucidation of the nature of the toxic agent and an endeavour to find some effectual means of combating it. Moebius [3] has made an antithyroid serum by using the blood of sheep from which the thyroid has been removed six weeks previously. This is administered to patients with Graves disease in doses of 5 c.c by mouth. Eight cases are reported by Hempel, Thienger, and Sainton, and

Pisanti, in which this treatment has been adopted, and in all of which, after the failure of other methods, marked improvement, amounting in some cases to practical cure, resulted. There seems to be no doubt that certain outlying parts of the human thyroid correspond to the parathyroids of certain mammals and of birds. And, further, it is probable that the parathyroid secretion is antitoxic to that of the thyroid. In animals in which the two structures are distinct, removal of the parathyroids produces tetany. And in human subjects the same symptoms often follow the total ablation of thyroid structures.[4] Moreover, it is clear that a relation exists between the activity of the thyroid secretion and the genital functions. Cretins are sexually immature and impotent, and Lorenz [2] has found that the removal of the thyroid in young animals is followed by sterility which is but little influenced by the subsequent use of thyroid substance. But, on the other hand, he refers to the case of a cretin, aged 28 years, in whom the sexual function was absent, but which was developed after the administration of thyroid extract; this man ultimately married, but had no issue. Crisafulli [2] reports favourably on the electrical treatment of goitre and Graves disease. The positive electrode is wrapped in wool soaked in iodine solution and placed over the gland, the negative pole being on some indifferent part of the body. Shrinkage of the thyroid followed, and there was a cessation of the nervous phenomena. Voss [3] finds that the serious toxic manifestation in acute Graves disease yield sometimes to the infusion of normal saline solution. In malignant disease of the thyroid little or nothing can be expected as a radical cure, but surgical treatment is often urgently demanded for the dyspnœa caused by the mechanical occlusion of, or pressure on, the trachea. Thompson [4] reports two such cases. One was a man of 29, the other a woman of 54. Dyspnœa was so urgent that the operation had to be done in the sitting posture, and, in the first case, under local anæsthesia only. After dividing the superficial structure, the isthmus of the thyroid is cut across, and then as much of the growth is rapidly shelled out as is practicable. A long tube must be passed down into the trachea, and in the first case it was necessary to use a piece of gas-fitter's lead pipe, 11 mm. in diameter, which was passed six inches down into the windpipe. In both cases the urgent and distressing dyspnœa was relieved until death occurred at intervals of two weeks and three months respectively.

[1] Amer. Jour. of Med. Sci., Feb., 1905. [2] Pract., May, 1905. [3] Edin. Med. Jour., April, 1905; Glasgow Med. Jour., April, 1905. [4] Edin. Med. Chron., May, 1905.

